# Effects of Fatigue and Unanticipated Factors on Knee Joint Biomechanics in Female Basketball Players during Cutting

**DOI:** 10.3390/s24144759

**Published:** 2024-07-22

**Authors:** Aojie Zhu, Shunxiang Gao, Li Huang, Hairong Chen, Qiaolin Zhang, Dong Sun, Yaodong Gu

**Affiliations:** 1 Faculty of Sports Science, Ningbo University, Ningbo 315211, China; 2311040119@nbu.edu.cn (A.Z.); 2211040050@nbu.edu.cn (S.G.); h1353472068@163.com (L.H.); 2 Doctoral School on Safety and Security Sciences, Óbuda University, 1034 Budapest, Hungary; chenhairong233@163.com (H.C.); zhangqiaolin123@gmail.com (Q.Z.); 3 Faculty of Engineering, University of Szeged, 6724 Szeged, Hungary

**Keywords:** fatigue, unanticipated factors, biomechanics, sidestep cutting, female basketball players

## Abstract

(1) This study examined the impact of fatigue and unanticipated factors on knee biomechanics during sidestep cutting and lateral shuffling in female basketball players, assessing the potential for non-contact anterior cruciate ligament (ACL) injuries. (2) Twenty-four female basketball players underwent fatigue induction and unanticipated change of direction tests, and kinematic and kinetic parameters were collected before and after fatigue with a Vicon motion capture system and Kistler ground reaction force (GRF) sensor. (3) Analysis using two-way repeated-measures ANOVA showed no significant interaction between fatigue and unanticipated factors on joint kinematics and kinetics. Unanticipated conditions significantly increased the knee joint flexion and extension angle (*p* < 0.01), decreased the knee flexion moment under anticipated conditions, and increased the knee valgus moment after fatigue (*p* ≤ 0.05). One-dimensional statistical parametric mapping (SPM1d) results indicated significant differences in GRF during sidestep cutting and knee inversion and rotation moments during lateral shuffling post-fatigue. (4) Unanticipated factors had a greater impact on knee load patterns, raising ACL injury risk. Fatigue and unanticipated factors were independent risk factors and should be considered separately in training programs to prevent lower limb injuries.

## 1. Introduction

Basketball is one of the most popular sports globally. As modern basketball continues to evolve, the level of physical confrontation has intensified, and the intensity of women’s basketball has gradually approached that of men’s basketball [1]. This phenomenon is associated with a high rate of lower limb joint injuries, including injuries to the knee and ankle. Non-contact anterior cruciate ligament (ACL) injury is a common knee injury among basketball players, often occurring during changes of direction, landing, and turning. Due to these rapid movements, basketball players face high risk of ACL injury, which can severely impact their sports careers. The ankle joint, a crucial component of the proprioceptive system, is also prone to injury [2], particularly during shooting movements. Cutting, which includes lateral shuffling and sidestep cutting, is a common change of direction in basketball. Studies have shown that approximately 70% of non-contact ACL injuries occur during changes of direction [3]. Video studies have also shown that most injuries occur during offensive cutting and defensive cutting [4]. The incidence of knee injuries varies by gender, age, and sport. And during basketball, female high school athletes injure their ACLs more often while jumping or landing (60%) than they do when participating in soccer (25%) [5]. Research has shown that in all sports, ACL injuries occur in 1 out of 29 female athletes, whereas the rate is 1 out of male athletes [6]. When the level of the sport is considered, ACL injuries occur at twice the rate in both amateur and elite females compared to males [6,7]. Although knee joint injuries occur at a higher rate in men overall, it has been found that women require surgery after knee injuries twice as often male athletes [8]. And the rate of ACL injuries in women’s basketball players is more than four times that in men’s basketball players. A study has found that there is a more significant ACL injury rate in females compared to males because females have a larger Q angle compared to males; it was previously believed that the Q angle impacts the lower extremity force line and increases the knee valgus moment in both static and dynamic scenarios, which can result in ACL injuries [9]. Additionally, during these movements, women tend to exhibit greater hip abduction, increasing their risk of ACL injury.

Many research teams are currently conducting comprehensive studies on the biomechanical characteristics and injury risk mechanisms related to side-cutting movements in basketball. Traditionally, experiments on cutting movements have been completed under anticipated conditions [10], with the characteristics of lower extremity movement being analyzed during the completion of the movement [11]. That is, the subjects complete the specified movements in a prepared state [12]. Consequently, several studies have conducted comparative analyses of lower extremity biomechanics during the lateral shuffle under both anticipated and unanticipated conditions. The results of Kim’s study found that compared with the expected conditions, the lateral component of the ground reaction force (GRF) increased, the flexion angle of the hip joint decreased, and the indexes of external rotation, internal rotation angle, and peak internal/external rotation moments of the knee and hip joints increased significantly when the lateral shuffle and sidestep cutting were completed under unanticipated conditions, which led to an increase in the risk of non-contact ACL injuries [13]. However, this did not fully reflect the real conditions in a game, where athletes have very little time to react, and most reactions occur under unanticipated conditions. Therefore, studying the biomechanics of lower limbs during lateral shuffling and sidestep cutting under both anticipated and unanticipated conditions is highly valuable.

In actual training and competition, fatigue is also considered a significant factor in reducing sports performance and causing injuries. Experimental research has shown that fatigue can reduce muscle strength and knee proprioception and increase joint laxity, ultimately resulting in decreased joint stability. Studies indicate that following fatigue-inducing activities, athletes may display heightened valgus and internal rotation angles, as well as increased internal/external valgus moments upon touchdown and during the deceleration phase when executing lateral and transverse cutting movements. These factors collectively elevate the likelihood of ACL injury occurrence [14]. Building on the above findings, both fatigue and unanticipated factors influence the biomechanical indicators of lower limb joints, such as the hip, knee, and ankle, during lateral shuffling and sidestep cutting movements. Some scholars have suggested that there is an interaction between fatigue and unanticipated factors [15], and some studies assert that the interaction between fatigue and unanticipated factors is not obvious and does not increase the risk of ACL injuries [14].

Therefore, some studies have comparatively analyzed the biomechanical differences in the lower limb joints during the completion of lateral shuffling and sidestep cutting, but there are no works in the literature analyzing the interaction or main effects of fatigue and unintended factors on the kinematics and kinetics of the hip, knee, and ankle joints in the lower extremities during turning movements, so as to investigate the biomechanical mechanisms of fatigue and unintended factors and their effects on non-contact ACL injuries during turning movements. There is an interaction between fatigue and unanticipated factors that affects the performance of lateral cutting movements and requires further investigation. Thus, our study aims to explore whether fatigue, unanticipated factors, and their interaction affect the biomechanics of lower limbs in female basketball players during lateral shuffling and sidestep cutting movements. This has important guiding significance for sports training and injury prevention. Through scientific research, new training methods and techniques can be developed to improve athletes’ competitiveness in competitions. For example, advanced motion capture technology and data analysis tools can be used to accurately measure and evaluate athletes’ movements, thereby providing personalized training suggestions.

## 2. Materials and Methods

### 2.1. Participants

A total sample of 24 women’s college basketball players (age: 23.4 ± 1.5 years; body mass: 57.3 ± 3.1 kg; height: 164.6 ± 2.4 cm) was analyzed by using G*Power 3.1.9.6 (Heinrich Heine University Düsseldorf, Germany) and criteria of 80% power, an alpha level of 0.05, and an effect size of 0.25 [16]. All subjects had more than 3 years of basketball experience. Subjects were all required to have had no lower limb injuries in the past year. Before the experiment began, the subjects were informed of the experimental content and signed the informed consent form after their consent was obtained. All participants had to sign a written informed consent form. The experiment was approved by the Ethics Committee of the University of Ningbo (RAGH20240310).

### 2.2. Sensors

For heart rate monitoring during the fatigue test, a Polar H9 heart rate sensor was used to monitor the subject’s heart rate changes in real time. This sensor can quickly respond to heart rate changes and is not affected by temperature or sweat. The heart rate sensor is made of soft textile material, providing comfortable wear and ensuring the accuracy of the experiment is not compromised [17,18]. 

The action data were captured using the Vicon sensor, a 3D motion capture system of 10 infrared cameras (Vicon Metrics Ltd., 10 MX-T20 cameras, Oxford, UK, frequency = 200 Hz). It was used to collect 38 reflective marked points attached to the subject; these points transmit the subject’s trajectory to the corresponding software. A three-dimensional ground force reaction sensor named Kistler (Kistler, Winterthur, Switzerland) was used to synchronously collect GRFs in three different directions with a sampling frequency of 1000 Hz. This can be reflected in the reaction force of the ground on the knee joint in both maneuvers. The captured kinetics and kinematics raw data of the whole body were imported into OpenSim 4.1 with the model Gait 2392 (Stanford, CA, USA), and the raw kinematics and kinetics were filtered and smoothed using a fourth-order Butterworth low-pass filter with cutoff frequencies of 20 Hz and 50 Hz, respectively [15]. The combination of these obtained data gave the motion of the subject’s knee joint in the horizontal, frontal, and sagittal planes.

### 2.3. Experiment Plan

This experimental program consists of two main components: the testing program and the fatigue program. All tests are conducted in two rounds. Initially, participants undergo both expected and unexpected lateral sliding as well as side-cutting action tests before reaching a state of fatigue. Subsequently, participants are fatigued based on a predefined fatigue protocol. Once participants meet the fatigue criteria, they repeat the same set of tests performed prior to fatigue. The test protocols remain consistent throughout both stages. The sequence of expected and unexpected tests for all participants is predetermined randomly.

#### 2.3.1. Test Plan

**Figure 1:**
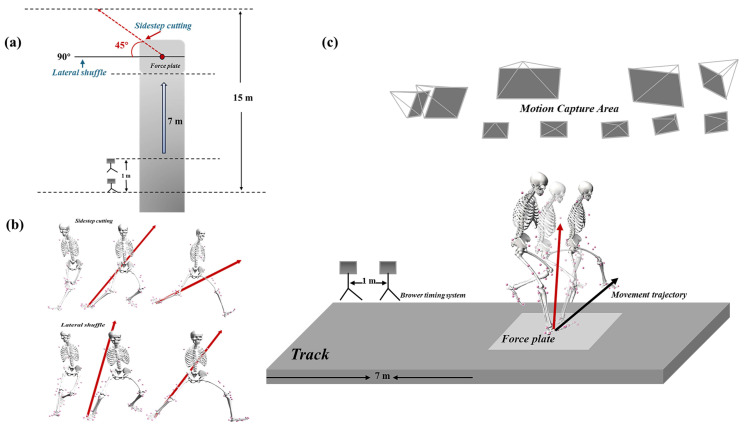
(**a**). Experimental procedure. (**b**) The types of sidestep cutting and lateral shuffling in this study. The red arrows represent the ground reaction forces. (**c**) Schematic diagram of the experiment. The red arrows represent the ground reaction forces. The black arrow represents the movement trajectory.

Before the experiment, the dominant leg (the kicking leg) of the subject was determined. The dominant leg of the subjects in this study was the right leg [6]. After this, the subjects were asked to warm up on the treadmill for 5 min at a warm-up speed of 8 km/h. After changing into the clothing for the experiment (tight-fitting clothing), they were briefed on the experimental procedures, test protocols, and safety measures, ensuring their familiarity with the experiment. Before the formal experiment began, 38 marks needed to be attached to the subjects to collect their static data. During the formal experiment, each subject was required to run at full speed on a 10 m field to complete the test under both anticipated and unanticipated conditions. A Brower timing system was placed on one side of the runway to determine the subject’s run-up speed. Because in basketball, 90° lateral shuffling and 135° sidestep cutting are often used in offense and defense [19], under anticipated conditions, the subjects were instructed to complete two actions: 90° lateral shuffling and 135° side-cutting [20] (Figures S1 and S2). Under unanticipated conditions, the subjects randomly performed either lateral shuffling or sidestep cutting based on the instructions issued by the experimenter and made sure their right leg was on the force platform. The experimenter provided specific instructions via a signboard, timing them to coincide with the moment the subject’s forefoot passed the front end of the photoelectric gate timer. Five valid data sets were collected for each condition, and three valid lateral shuffling and sidestep cutting actions were selected for analysis under both anticipated and unanticipated conditions (Figure 1). 

The actions selected in this study are all common change of direction actions in actual basketball games. Athletes needed to reflect these actions using their vision, simulating unanticipated situations in basketball games. This allowed the randomly completed side-cutting actions to meet the unanticipated assumptions to the greatest extent.

#### 2.3.2. Fatigue Protocol

The choice of fatigue program comes from previous studies, using heart rate assessment or the Borg Fatigue Rating Scale. The changes in the subject’s heart rate are detected by a wearable heart rate monitor. The maximum heart rate × 80% = (220—age) × 80% [21]. The subjects were asked to perform a 300-m sprint shuttle run [22], 10 × 30-m sprints, and 30 s of passive rest between each run (Figure S3). Combined with the subject’s heart rate, after the 300-m sprint, the subject used a subjective scale to determine their degree of fatigue, and then continued to repeat the sprint until their subjective scale score reached 17 points [21]. The time that it took subjects to reach fatigue was 15.47 ± 1.83 min in this study. After the fatigue experiment, the subjects immediately entered the laboratory for a new round of experiments under anticipated and unanticipated conditions to ensure the accuracy of the experimental results [23].

### 2.4. Data Analysis

The initial stage of landing during a cutting action was usually considered to be a susceptible stage for knee joint injury. In addition, Liu’s study has shown that the peak load on the ACL occurs at the first peak of the GRF [24]. Our study determined the moment of touchdown based on the data from a three-dimensional force platform. The lateral ground reaction force (GRF ≥ 20 N) was defined as the ground reaction force component in the same direction as the lateral cutting direction (LGRF); the vertical ground reaction force was the ground reaction force component in the same vertical direction (VGRF). The horizontal backward reaction force of the ground was the ground reaction force component in the same posterior horizontal backward direction (PHGRF). The joint kinetic data were adjusted based on each participant’s body mass. Using MATLAB version 2019b (The MathWorks, Natick, MA, USA), the joint kinematic and kinetic data were time-normalized to the stance phase, consisting of 101 data points per stance phase [23]. 

The selected indicators include the first peak of the lateral GRF; the first peak of the vertical GRF; the first peak of the horizontal backward GRF; the sagittal plane angle values of the hip, knee, and ankle joints corresponding to the IC moment (IC is defined as the moment of ground contact); the sagittal plane angle values of the three joints corresponding to the first peak moment of LGRF; the moments of the knee joints in the sagittal plane; and the sagittal plane range of motion of the knee joints. According to previous studies, the flexion and extension of the hip, knee, and ankle joints were defined as negative (−) and positive (+), respectively. All indicators were calculated and exported through OpenSim. The trajectory of the marked points and the ground reaction data collected during the experiment were processed and converted using a custom MATLAB program. Following the published scheme, the OpenSim workflow was implemented. Initially, the weights of the marked points in the model were manually adjusted. The model was then scaled to match the participants’ anthropometric characteristics, ensuring that the root mean square error between the marked points and the virtual marked points in the experiment was less than 0.02 m. The maximum error was less than 0.04 m. Second, the inverse kinematics algorithm was applied to determine the joint angles that minimized the error between the marked points and the virtual marked points in the experiment. Subsequently, the inverse dynamics algorithm was utilized to calculate the joint moments [25,26]. 

### 2.5. Statistical Analysis

The data results were expressed as mean ± standard deviation (M ± SD). A two-way repeated-measures analysis of variance (ANOVA) was used (pre-fatigue × post-fatigue) to test the differences between two groups (anticipated vs. unanticipated conditions) and to assess whether there was any interaction between anticipation and fatigue. SPSS27 (SPSS, Inc., Chicago, IL, USA) was used as the statistical software, and the significance level α was set to 0.05. One-dimensional statistical parametric mapping (SPM1d) was also used to analyze the moments of the knee joint, and relied on random vector field theory to account for data variability [27]. The statistical analyses were conducted in MATLAB R2023a (The Math Works, Natick, MA, USA).

## 3. Results

### 3.1. Kinematics

The kinematic findings in our study revealed that the subjects’ knees were in different states during the IC moments in the lateral shuffle movement, and the subjects showed increased internal rotation angles during the IC moments in the unanticipated conditions compared to the anticipated conditions (*p* < 0.05); no significant difference was seen in the inversion/eversion angles, and the fatigue effect resulted in greater inversion and eversion angles for both the lateral shuffle and the sidestep cutting movements (*p* < 0.05). The interaction between fatigue and the anticipation effect was not significant (*p* > 0.05) (Table 1 and Table 2).

### 3.2. Kinetics

**Figure 2:**
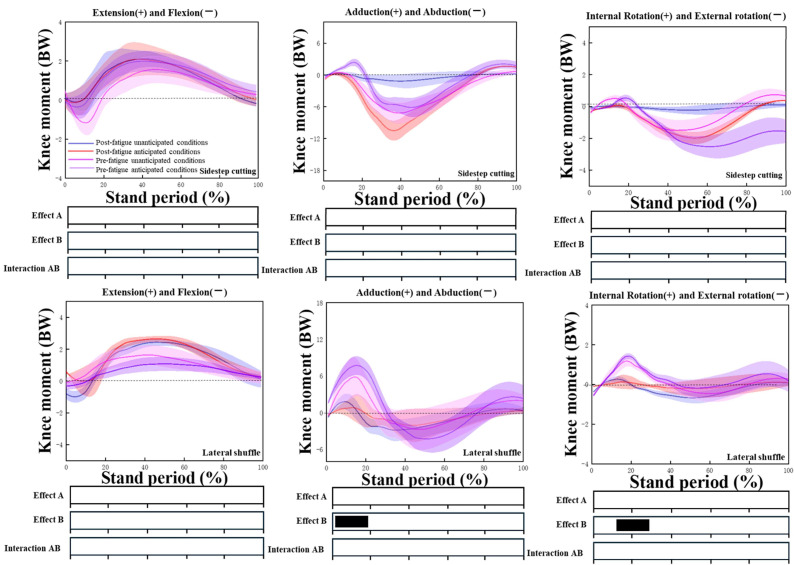
Knee joint moment in sagittal, frontal, and transverse planes during the stance phase in sidestep cutting and lateral shuffling. The black dashed line represents a value of 0. Effect A is fatigue, effect B is unanticipated factors, and significant main effects (*p* < 0.05) are black horizontal bars at the bottom of the figure during corresponding periods from SPM1d analyses.

The results for the kinetics in our study indicated that, in the unanticipated conditions, the knee joint moments in the frontal and horizontal planes of the subjects shifted dramatically from inversion and internal rotation moments to eversion and external rotation moments more quickly compared to the anticipated conditions during lateral shuffling and sidestep cutting movements. The knee flexion moment, adduction moment, and rotation moment indexes corresponding to the first peak value of LGRF and its peak moment were significantly different (*p* < 0.05), but the VGRF and PHGRF were not significantly different (*p* > 0.05), and the interaction between fatigue and unintended effects was not significant (*p* > 0.05) (Figure 2, Table 3).

**Figure 3:**
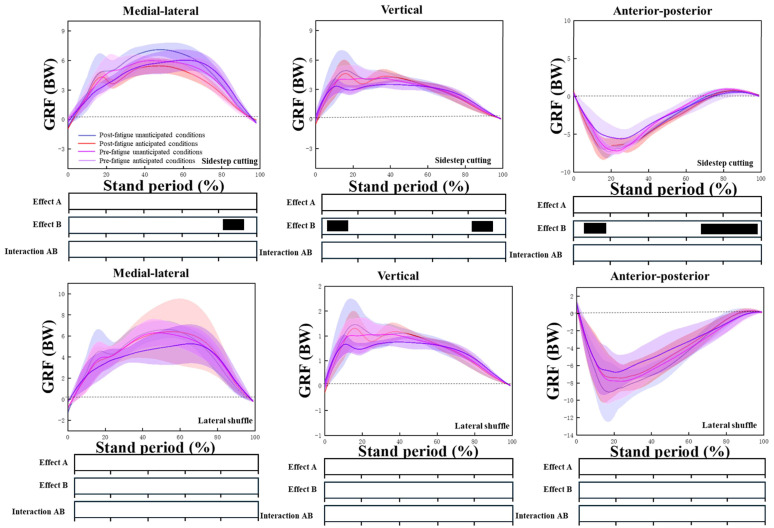
The ground reaction force curves during sidestep cutting and lateral shuffling. The black dashed line represents a value of 0. Effect A is fatigue, effect B is unanticipated factors, and significant main effects (*p* < 0.05) are black horizontal bars at the bottom of the figure during corresponding periods from SPM1d analyses.

According to the analysis results of SPM1d, there was no interaction between anticipated and fatigue conditions, and there were significant differences in the LGRF (*p* < 0.001) (85–100%), VGRF (*p* < 0.001) (5–15%, 85–100%), and PHGRF (*p* = 0.002) (0–20%,60–90%) for sidestep cutting. There were also some differences in the eversion moments and rotation moments of the knee joint during the lateral shuffle (Figure 3).

## 4. Discussion

The findings of this research emphasize the importance of fatigue and unanticipated factors as high-risk contributors to lower limb injuries in basketball players. The results of our study showed that fatigue and unanticipated factors affected knee kinematic and kinetic indexes during the lateral shuffle and sidestep cutting to different degrees, respectively, whereas fatigue and unanticipated factors did not interact. The following discussion will focus on the fatigue–unanticipated factor interaction, the fatigue effect, and the unanticipated factor effect.

### 4.1. Interaction between Fatigue and Unanticipated Factors

The results of this study indicated that there were no interactions between the factors of fatigue and unanticipated events. Kahlid and Collin [14,28] analyzed subjects completing lateral shuffling and sidestep cutting under unanticipated conditions after fatigue, revealing no increase in knee loading, and the study concluded that the combination of both fatigue and unanticipated factors did not additionally increase the risk of ACL injury, consistent with the results of our study. But Mclean [29] and Borotikar [15] indicated in their findings that, when participants landed on one foot in front of the other under unanticipated circumstances after completing the fatigue program, there was a significant increase in the peak knee valgus angle. This suggests an interaction between fatigue and unanticipated factors. In this scenario, the likelihood of ACL injury is higher when these two factors interact, compared to when they act individually. Some findings of the present study are consistent with these results. However, most kinematic and kinetic indicators show that there is no interaction between fatigue and unanticipated factors. We believe that the possible reasons for this difference include the difference in the form of the movements studied; this study and Khalid’s [28] research addressed assisted sidestep cutting and lateral shuffling in the frontal plane, whereas the jumping and landing maneuvers used in Mclean’s study mainly showed movements in the sagittal plane.

We believe that the lack of interaction between fatigue and unanticipated factors in side-cutting movements can be explained by the difference in action strategy selection. Fatigue and unanticipated factors lead to different degrees of action selectivity differences, which are important for the completion of actions. When two adverse factors are combined, the human body perceives and predicts the risk of injury more effectively, leading to a change in movement strategy. It is not difficult to see that fatigue and unanticipated factors still have a significant impact on lateral shuffling and sidestep cutting movements.

### 4.2. Fatigue Effects

The results showed that by implementing the fatigue procedure of this study, a significant decrease in the knee inversion moment after fatigue under expected conditions and a significant increase in the eversion moment after fatigue under unanticipated conditions occurred. No significant fatigue effects were observed on knee kinematics and kinetics in the sagittal and horizontal planes, nor were there notable changes in the components of the GRF. This aligns with the findings of McLean. This is because the increased knee eversion moment in fatigue indicates a decrease in the ability of the musculoskeletal system to maintain joint stability [30].

However, it has been shown that fatigue increases the inversion moment of the knee. Our study suggested that fatigue increases eversion moments and rotation moments at the knee joint. Different fatigue protocols and the selection of indicators at various phases are likely responsible for the discrepancies between the results of these studies. In terms of kinematics, our studies show that fatigue increases the internal rotation angle of the knee [15]. Although the kinematics and kinetics of the knee during lateral shuffling and sidestep cutting were affected by the different fatigue protocols, the overall fatigue effect was still apparent. This may be attributed to the body’s reduced ability to control posture after fatigue, thereby affecting the stability of the knee joint.

### 4.3. Unanticipated Effects

It can be found that the first peak values of the GRFs in the vertical and horizontal directions did not differ statistically between the two conditions compared to the side-cutting maneuver in the anticipatory condition. However, the difference in the first peak values of the lateral component was significant. This is because, in the unanticipated conditions, subjects had to make rapid postural adjustments to complete the side-cutting maneuver shortly after receiving a visual change of direction command. This relatively “hasty” bracing action led to insufficient cushioning, significantly increasing the impact force in the direction of frontal plane lateralization [31].

Most of the results of our study showed that, compared with the anticipated conditions, the unanticipated conditions had a more significant effect on the kinematics of the knee joints at the moment of contact and the first peak of the lateral GRFs. Compared to the anticipated conditions, the knee in the unanticipated conditions exhibited greater flexion, adduction, and rotation angles, as well as greater knee adduction and flexion moments. These findings are generally consistent with those of previous researchers [32]. Some studies suggested that in unanticipated conditions, subsequent lateralization may only be achieved by utilizing an outward landing of the supporting foot as well as an outward tilt of the body [33]. This also increases the valgus and internal rotation angles of the knee upon landing, a change that may elevate the risk of non-contact ACL injuries. An excessive knee valgus angle weakens the ligamentous constraints of the knee joint, thereby increasing the load on the knee joint [34]. The greater the angle of internal rotation of the knee, the more likely the ACL is to rupture.

This study’s results show that the maximum knee flexion angle during the braking phase under unanticipated conditions was greater than that under anticipated conditions, which is consistent with Borotikar et al. [15]. This study suggests that the knee flexion angle during the stance period of the cutting action under unanticipated conditions was greater than that under anticipated conditions. This may be because during the run-up under unanticipated conditions, the subjects need to pay more attention to the action instructions given by the sign to determine the correct action. When they receive the action instructions, to complete the side-cutting or lateral shuffle action faster, their preparations for the braking and extension phases are not sufficient compared to those in the anticipated conditions, and the knee flexion angle is significantly greater than the value under the anticipated conditions. Some studies have suggested that increased knee flexion limits the force of the quadriceps, which will cause the posterior femoral muscles to limit the anterior displacement of the tibia [35]. This result seems to suggest that the risk of ACL injury is reduced under unanticipated conditions. However, the results of this study show that when completing the side-cutting action under unanticipated conditions before and after fatigue, the knee flexion angle at the time of contact was less than 30 degrees. Some studies have shown that most non-contact ACL injuries occur when the sagittal knee flexion angle is less than 30 degrees [3]. Therefore, it can be determined that the size of the sagittal knee flexion angle is a high-risk factor for ACL injuries.

The results of the kinetic research showed that compared with the anticipated conditions, there was a big difference in the first peak value of the lateral component GRF during the side-cutting action under the unanticipated conditions. This was because under unanticipated conditions, the influence of the subject’s visual instructions requires different actions to be completed in a very short time, which leads to insufficient braking of the subject’s support actions. Park et al. [36] believed that the increase in lateral GRF during the lateral cutting action under unanticipated conditions is due to the increase in hip abduction and the trunk lateral flexion angle. When the subject completes the lateral cutting action, the supporting leg moves with greater force. The force of hitting the ground promotes a change of direction, which puts the human body in a position more susceptible to injury. Moreover, the increase in lateral GRF induces increased contraction of the quadriceps muscle and increased muscle strength, causing the ACL to bear a higher load [24].

Consideration should be given to some limitations of the current study. This experimental study conducted in a laboratory setting may not fully replicate the fatigue levels and stimulus responses experienced by athletes in real-life competition scenarios. In future research, we will strive to delve into real competition settings and explore the impact of fatigue and unanticipated factors on athletes’ lower limb biomechanics through more diverse methods. In addition, the instruments used (such as the marker infrared reflective balls placed on the subjects) may have affected the subjects’ performance during fatigue intervention and change of direction movements. The tights provided in the experiment were designed to prevent the reflective dots from shifting and causing inaccurate experimental data. They are different from the loose clothing used in competition. Future research could address these limitations by comparing athletes from different groups, thereby further studying the mechanisms of knee injuries in female athletes.

## 5. Conclusions

Our study delved into the interplay between fatigue and unanticipated factors during lateral shuffling and sidestep cutting among female basketball players. While some research suggested that knee loads do not significantly rise when both fatigue and unanticipated factors are at play, others indicate a potential interaction, heightening the risk of ACL injury. However, most indicators showed no such interaction, likely stemming from variances in movement form and strategy influenced by fatigue and unanticipated factors. Nonetheless, both fatigue and unanticipated conditions independently affect knee kinematics and kinetics, with the fatigue protocol significantly shaping study outcomes. Fatigue compromises postural control and impacts lower limb joint stability, while unanticipated conditions prompt swift adjustments, elevating the ACL injury risk. These findings underscore the importance of integrating both factors into athletes’ injury prevention strategies, particularly in demanding sports like basketball.

The interaction between fatigue and unanticipated factors in lateral movements is not significant. The simultaneous interaction of fatigue and unanticipated factors may not increase the risk of ACL injury compared to either factor alone. Compared with fatigue factors, unanticipated factors change the load pattern of the knee joint during side-cutting movements, which may lead to an increased risk of non-contact ACL injury. Fatigue and unanticipated factors could be considered as two independent risk factors in the design process of training to prevent lower limb joint injuries.

## Figures and Tables

**Table 1:** Angles of knee joint in sagittal, frontal, and horizontal planes at touchdown moment.

Variables (°)	Pre-Fatigue		Post-Fatigue		Fatigue	Anticipated	Fatigue × Anticipated
	Anticipated	Unanticipated	Anticipated	Unanticipated			
Knee flexion angle (sidestep cutting)	−28.25 (5.68)	−26.38 (3.60)	25.31 (7.76)	−26.25 (6.58)	0.554	0.936	0.52
Knee adduction angle (sidestep cutting)	1.2 (0.76)	0.89 (1.79)	4.76 (4.19)	3.98 (1.05)	0.582	0.457	0.165
Knee rotation angle (sidestep cutting)	−1.13 (1.77)	−3.80 (1.63)	−9.03 (1.68)	−6.50 (1.32)	0.388	0.026 *	0.953
Knee flexion angle (lateral shuffle)	−26.84 (5.73)	−26.52 (6.49)	27.07 (5.27)	−26.52 (6.49)	0.956	0.811	0.95
Knee adduction angle (lateral shuffle)	1.21 (2.65)	4.35 (1.34)	1.76 (1.63)	−0.52 (1.23)	0.024 *	0.457	0.582
Knee rotation angle (lateral shuffle)	−7.68 (1.17)	−3.50 (0.82)	10.37 (2.21)	−11.74 (1.65)	0.091	0.014 *	0.581

Note: The bold and “*” represent significant differences, with *p* < 0.05.

**Table 2:** Angle ranges of the knee joint in the sagittal, frontal, and horizontal planes at the first lateral peak.

Variables (°)	Pre-Fatigue		Post-Fatigue		Fatigue	Anticipated	Fatigue × Anticipated
	Anticipated	Unanticipated	Anticipated	Unanticipated			
Knee flexion angle (sidestep cutting)	−36.23 (10.76)	−32.17 (7.36)	−35.88 (5.86)	−33.97 (9.87)	0.864	0.188	0.624
Knee adduction angle (sidestep cutting)	−3.3 (3.52)	0.87 (1.46)	−0.16 (3.06)	−0.73 (3.13)	0.381	0.015 *	0.058
Knee rotation angle (sidestep cutting)	−4.07 (9.97)	−9.37 (3.97)	−9.95 (3.36)	−8.21 (3.26)	0.284	0.034 *	0.259
Knee flexion angle (lateral shuffle)	−32.92 (7.64)	−40.35 (8.98)	−32.86 (4.86)	−38.65 (8.34)	0.279	<0.01 *	0.949
Knee adduction angle (lateral shuffle)	−6.94 (1.10)	−1.18 (4.78)	0.22 (3.08)	−3.40 (2.24)	0.043 *	<0.01 *	0.344
Knee rotation angle (lateral shuffle)	4.30 (3.44)	−3.82 (1.86)	−4.82 (2.69)	−5.40 (3.63)	0.104	0.02 *	0.189

Note: The bold and “*” represent significant differences, with *p* < 0.05.

**Table 3:** Peak ground reaction force (BW) and knee joint moments at the first peak for sagittal, frontal, and horizontal plane ground reaction forces.

Variables	Pre-Fatigue		Post-Fatigue		Fatigue	Anticipated	Fatigue × Anticipated
	Anticipated	Unanticipated	Anticipated	Unanticipated			
LGRF first peak/BW (N·m·kg^−1^) (sidestep)	3.85 (1.78)	4.05 (2.04)	5.05 (2.05)	4.92 (2.68)	0.106	0.002 *	0.819
VGRF first peak/BW (N·m·kg^−1^) (sidestep)	3.78 (0.70)	4.84 (0.90)	5.23 (1.15)	6.06 (1.72)	**<0.01** *	0.022	0.756
PHGRF first peak/BW (N·m·kg^−1^) (sidestep)	−5.22 (1.55)	−5.82 (3.64)	−6.89 (2.66)	−6.56 (5.09)	0.277	0.884	0.620
LGRF first peak/BW (N·m·kg^−1^) (shuffle)	3.58 (1.42)	4.15 (1.88)	4.01 (2.23)	5.90 (1.65)	0.094	0.035 *	0.237
VGRF first peak/BW (N·m·kg^−1^) (shuffle)	3.94 (0.81)	4.75 (0.74)	5.45 (0.90)	6.09 (1.27)	**<0.01** *	0.057	0.805
PHGRF first peak /BW (N·m·kg^−1^) (shuffle)	11.50 (19.02)	−6.67 (2.96)	−7.19 (3.25)	−9.20 (4.08)	0.775	0.307	0.671
Flexion moment (N·m·kg^−1^) (sidestep)	−3.45 (0.34)	−0.09 (0.45)	0.18 (1.37)	0.76 (1.66)	0.339	0.331	0.658
Adduction moment (N·m·kg^−1^) (sidestep)	4.02 (1.94)	−2.08 (2.35)	−0.99 (1.97)	−0.56 (0.72)	0.013 *	0.006 *	0.066
Rotation moment (N·m·kg^−1^) (sidestep)	2.92 (3.95)	0.37 (0.71)	0.31 (1.12)	−0.04 (0.09)	0.284	0.034 *	0.259
Flexion moment (N·m·kg^−1^) (shuffle)	−0.03 (0.32)	1.25 (0.47)	0.65 (0.52)	−0.01 (0.37)	0.080	<0.01 *	0.151
Adduction moment (N·m·kg^−1^) (shuffle)	3.48 (1.86)	4.20 (2.89)	−0.04 (1.4)	0.32 (1.63)	0.708	0.003 *	0.904
Rotation moment (N·m·kg^−1^) (shuffle)	0.88 (0.54)	0.92 (0.90)	0.22 (0.25)	−0.05 (0.46)	0.909	0.006 *	0.949

Note: The bold and “*” represent significant differences, with *p* < 0.05.

## Data Availability

The data that support the findings of this study are available on reasonable request from the corresponding author. The data are not publicly available due to privacy or ethical restrictions.

## Supplementary Materials

The following supporting information can be downloaded at: https://www.mdpi.com/article/10.3390/s24144759/s1, Figure S1: A lateral shuffle schematic diagram, Figure S2: A side-step cutting schematic diagram, Figure S3: Fatigue intervention diagram.
