# Supplementary material for: Effects of Fatigue and Unanticipated Factors on Knee Joint Biomechanics in Female Basketball Players during Cutting
Source: Sensors (Basel). 2024 Jul 22;24(14):4759. doi: 10.3390/s24144759 (PMC11280919; doi:10.3390/s24144759)
Supplement: Supplementary file 1 [file sensors-24-04759-s001.zip › sensors-3092840-supplementary.pdf]

Effects of fatigue and unanticipated factors on knee joint bio-mechanics in female basketball players during cutting (Supplemente materials)

Lateral shuffle is one of the experimental movements. A Vicon 3D infrared sensors with ten cameras is used to capture the subject's movement trajectory (kinematic data), and a Kistler 3D force platform is used to collect the subject's kinetics data. When the ground reaction force that can be collected on the force platform is greater than 20N, the entire standing period begins. All movements involving force have been standardized by weight. The subjects in the laboratory quickly run across the calibrated track and make a 90° cross-section to the left at the moment when the main leg steps on the force platform.

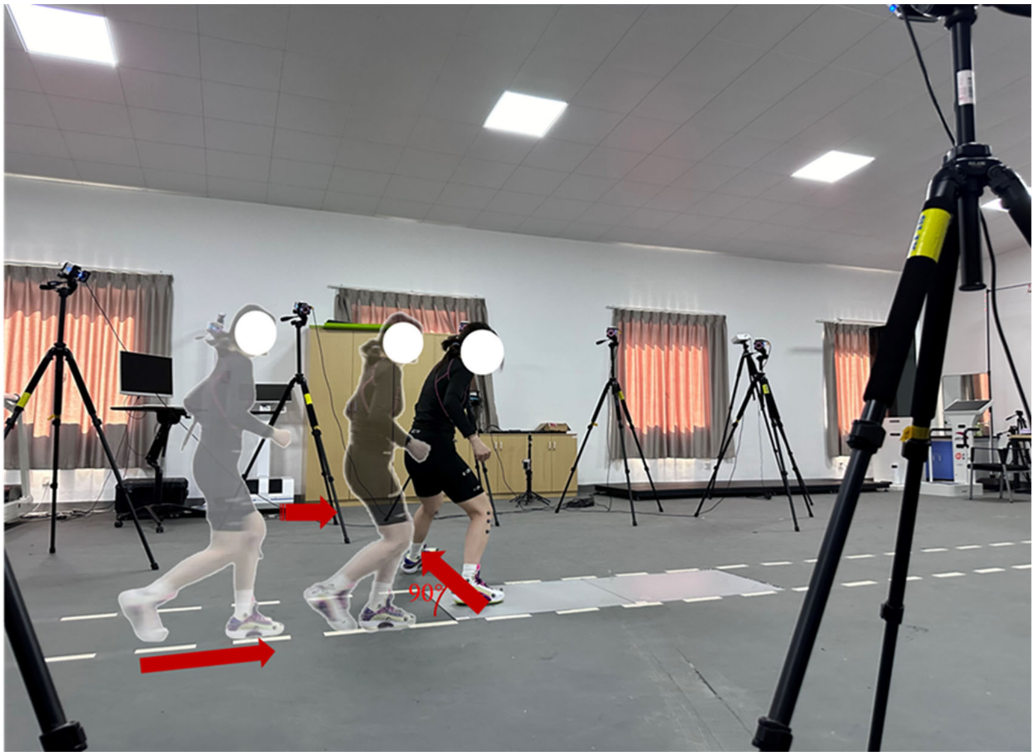

Figure S1. A lateral shuffle schematic diagram.

Another action in the experiment is side-step cutting. Its action collection process and experimental device are the same as lateral shuffle. The difference between the two is the angle when leaving the force platform. When completing the side-step cutting action, the subject moves 135° upward to the left at the moment when the

dominant leg leaves the force platform.

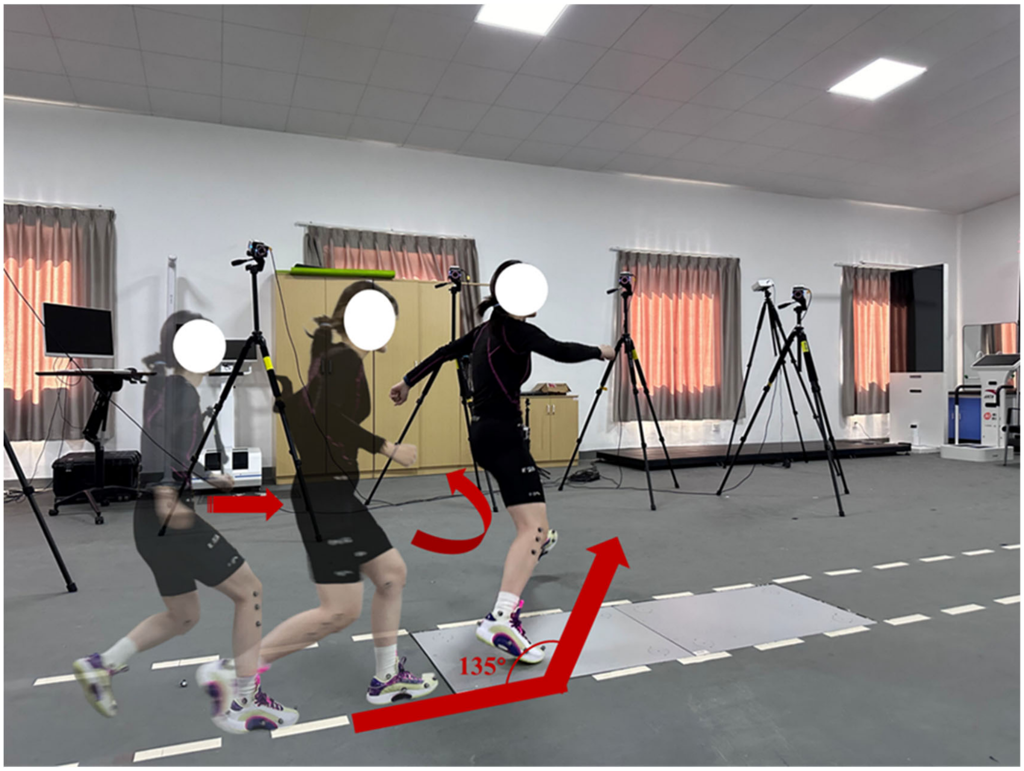

Figure S2. A side-step cutting schematic diagram.

We conducted fatigue intervention in another space with a 30-meter running track. The test plan simulated the most common sprint and return run in basketball games. This is also one of the common methods in basketball training. The subject touched the first cone barrel with her hand to indicate the start, then sprinted at full speed on the track, and touched the second cone barrel again when she reached it, and the one-way trip ended. There was a 10-second rest time in the middle of a round trip. Until the subject's various indicators reached the set fatigue standard.

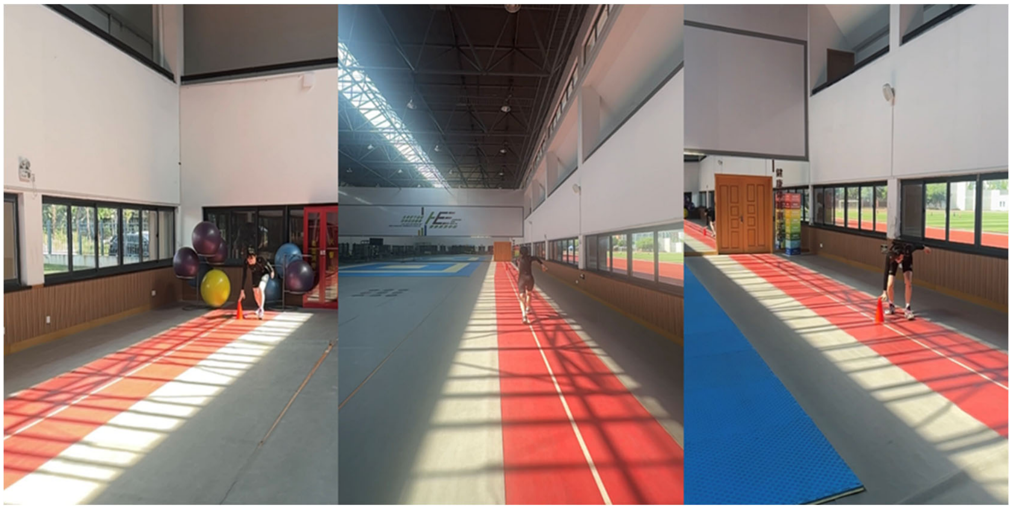

Figure S3. Fatigue intervention diagram
